# Effects of Maternal Separation and Subsequent Stress on Behaviors and Brain Monoamines in Rats

**DOI:** 10.3390/brainsci13060956

**Published:** 2023-06-15

**Authors:** Polina V. Mavrenkova, Nadezhda N. Khlebnikova, Irina B. Alchinova, Marina S. Demorzhi, Batozhab B. Shoibonov, Mikhail Yu. Karganov

**Affiliations:** 1 Institute of General Pathology and Pathophysiology, 8 Baltiyskaya St., 125315 Moscow, Russia; p-vottak@yandex.ru (P.V.M.); nanikh@yandex.ru (N.N.K.); alchinovairina@yandex.ru (I.B.A.); demorji@yandex.ru (M.S.D.); 2 P. K. Anokhin Institute of Normal Physiology, 8 Baltiyskaya St., 125315 Moscow, Russia; shoibonov@mail.ru

**Keywords:** rat, maternal separation, hyperactive phenotype, monoamines in brain structures

## Abstract

Childhood adversity can induce maladaptive behaviors and increase risk for affective disorders, post-traumatic stress disorder, personality disorders, and vulnerability to stress in adulthood. Deprivation of maternal care interrupts brain development through the disturbance of various neurotransmitters, however, the details remain unclear. The features of the symptoms of disorders are largely determined by early stress protocol, genetic characteristics (line), and the sex of the animals. The purpose of current study was (1) to assess behavioral changes in adult Wistar rats of both sexes after early life stress; (2) to determine the levels of monoamines in brain structures involved in the motor, emotional, and social reactions in rats aged 1 and 2 months; and (3) to determine the level of monoamines after physical or emotional stress in adult rats. The rat pups were separated from their dams and isolated from siblings in tight boxes at a temperature of 22–23 °C for 6 h during postnatal days 2–18. The data were processed predominantly using two-way analysis of variance and the Newman–Keys test as the post hoc analysis. The adult rats demonstrated an increase in motor activity and aggressiveness and a decrease in levels of anxiety and sociability. Behavioral disturbances were accompanied by region-, sex-, and age-dependent changes in the levels of monoamines and their metabolites. The dopaminergic and noradrenergic systems were found to be sensitive to psycho-emotional stress.

## 1. Introduction

Traumatic experiences are not uncommon in life. The term “complex trauma” refers to traumatic events that are chronic, invasive, and primarily interpersonal (e.g., child abuse and neglect by primary caregivers) [1]. These long-term childhood adversities are associated with impairment of brain development and an increased risk of mental disorders such as post-traumatic or complex post-traumatic stress disorder (PTSD, CPTSD) and borderline personality disorder (BPD) in adults [2,3,4]. Complex trauma results in emotional dysregulation, interpersonal problems, and maladaptive coping behavior, including suicidality [5,6]. Clinical observations show that CPTSD and BPD have overlapping symptoms of anxiety sensitivity, vulnerability to psychological stress, dissociation, and relationship problems arising from insecure attachment [7,8]. Empirical evidence suggests that BPD only accounts for a limited portion of the symptoms reported by survivors of childhood trauma, and that characteristics of CPTSD and BPD are correlated with several other psychiatric disorders (for example, depression, bipolar disorder, and substance abuse), and, at the same time, differ from them [6,9]. A hypothesis was advanced positing that PTSD, cPTSD, and BPD may represent a continuum based on either a sequential progression over time or a hierarchy of levels of psychopathological severity [10]. The findings suggest that PTSD and CPTSD often occur in the absence of BPD symptoms, but that BPD more often occurs with comorbid PTSD and CPTSD symptoms than alone. This being said, there is a subset of BPD-diagnosed persons who have no history of childhood maltreatment or other trauma exposure. On the other hand, not all people having traumatic antecedents beginning in childhood develop mental disorders. However, the mechanisms that determine vulnerability to early life stress, the developmental trajectories of psychopathology, and neurobiological markers associated with certain symptoms of childhood trauma-induced disorders are poorly understood. In particular, clinical studies of monoamines—norepinephrine (NE), dopamine (DA) and serotonin (5-hydroxytryptamine, 5-HT)—in PTSD/CPTSD/BPD have been limited.

The monoamine systems have a great impact on memory, cognition, and emotions [11]. Studies with administration of monoamine reuptake inhibitors to healthy men showed that methylphenidate, which elevates DA and NE content in both the cortex and subcortical structures, increased the number of correct responses during the visual task, while atomoxetine, which mainly affects the frontal cortex, led to more impulsive reactions, and citalopram increased the number of missed stimuli [12]. The monoamines are involved in the implementation of impulsive and aggressive behavior, emotional instability, inhibitory control of these behavioral patterns, and a number of other symptoms of disorders associated with traumatic experiences [13,14,15,16,17,18,19,20,21,22,23,24]. Regional and gender specificity of 5-HT system activity in mediating such symptoms of BPD as impulsive aggression and emotional dysfunction were recently reported [14,18,21,23]. An interesting suggestion has been made about how the interaction of 5-HT and NE systems can determine the aggression pattern [25]: low activity of the 5-HT system in combination with high activity of the NE system can lead to impulsive outward directed aggression, whereas low activity of 5-HT and low activity of NE can lead to self-directed impulsive aggression.

Significant progress in understanding the neurochemical mechanisms involved in the development of long-term impairments in response to traumatic experiences can be achieved through animal modeling. The large list of PTSD symptoms, many of which can be defined only in a subjective manner [26], make it challenging to develop suitable animal models of the disorder. Traditionally, animal models are used that recapitulate certain behavioral features of human PTSD. One commonly used behavioral paradigm adopted to assess PTSD-like symptoms in animal model is early life stress, above all, the stress of maternal separation in the neonatal period [27,28,29,30,31]. Some researchers believe that this method could be used to model in rodents a range of BPD symptoms [32,33], for instance, aggression [34], disturbances in social behavior [35], and impulsivity [36]. Adult animals subjected to maternal deprivation during early ontogeny demonstrated anxiety-like [37] and/or depressive-like behavior [38]. The development of reduced anxiety is less frequently reported [39,40]. The result can be influenced by various factors, including weaning protocol, age, sex of animals, and species/line of rodents [31,41,42].

Studies on rodent models of early stress have revealed behavioral disorders in adult animals and changes in the monoamine levels in several brain regions associated with abnormal behavior. The separation of rat pups from their mother on postnatal day 1–21 (P1–P21) increased DA turnover in the prefrontal cortex and hippocampus of females and 5-HT turnover in males. In addition, an increase in the NE turnover in the striatum was observed in females and a decrease in DA turnover in the hippocampus in males [43]. In another study, prolonged maternal separation of rat pups on P5–P20 caused an increase in the DA level in the striatum and reduced DA turnover in the prefrontal cortex in females, while males had reduced levels of 5-HT in the prefrontal cortex; the level of 5-HT in the hippocampus was decreased in rats of both sexes [44]. Thus, the results are contradictory, and further studies in this field are required.

According to modern understandings of the pathogenesis of mental disorders, childhood adversity creates predisposition to psychopathology, which can manifest itself after repeated stressful exposure in adult age (the double-hit hypothesis) [30]. In model experiments, maternal deprivation is often used as the first stressful effect in early ontogeny and at a later age, the animal is again exposed to acute or chronic stress [45]. However, monoaminergic systems were practically not studied in these models. For instance, Novac et al. [45] reported that postnatal maternal separation and immobilization stress during puberty have a synergistic effect on the expression of D2 receptors in the striatum. In another study, the rats were subjected to early-life maternal separation stress and the repeated stress of corticosterone administration during adulthood. The expression of DA receptors was studied in the medial prefrontal cortex, caudate nucleus, and nucleus accumbens and an increased number of these receptors was found in the nucleus accumbens [46].

Considering that the weaning protocol can influence the pattern of behavioral disorders associated, among others, with a change in the functioning of monoamine neurotransmission, the aim of the present study was (1) to assess behavioral changes in adult young rats of both sexes after the early stress of maternal and sibling separation; (2) to determine the levels of monoamines in brain structures presumably involved in the formation of emotional and social disorders in rats aged 1 and 2 months; and (3) to determine the levels of monoamines after repeated stress in adult rats.

## 2. Materials and Methods

### 2.1. Animals

The study was performed on Wistar rats bred at the vivarium of the Research Institute of General Pathology and Pathophysiology (Mercury system, registration number: RU 1487336). Pregnant and lactating females and later animals of the experimental and control groups were housed in controlled room temperature and humidity conditions (22 ± 1 C and 55 ± 25%) with natural lighting and free access to water and food (balanced pelleted feed for rodents, Laboratorkorm LLC, Russia, Moscow). During the experiment, the health status of the animals was monitored by a veterinarian. All manipulations with animals were carried out under supervision of the Ethical Committee of the Research Institute of General Pathology and Pathophysiology (approval protocol No. 2-22 dated 12 May 2022) in accordance with Directive 2010/63/EU of the European Parliament and of the Council “On the protection of animals used for scientific purposes”, and Guide for the Care and Use of Laboratory Animals: Eighth Edition, 2011.

### 2.2. Study Design

**Figure 1:**
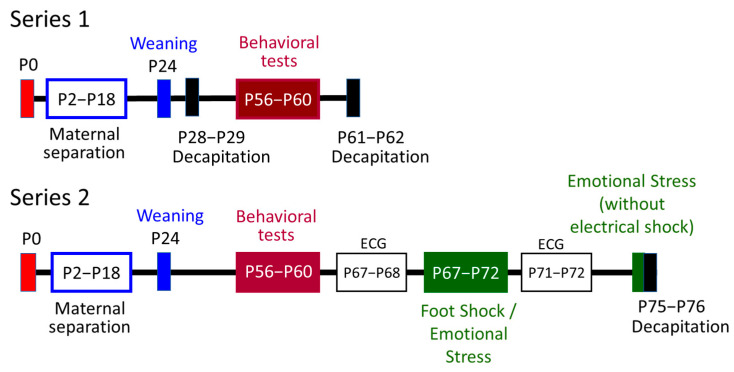
Study design. Red rectangles: day of birth, postnatal day 0 (P0); P2–P18: maternal separation and sibling isolation in the experimental group; blue rectangles: weaning, P24; brown rectangles: behavioral tests, P56–P60; green rectangle: additional stress modeled by exposure to foot shock or witnessing cage mates subjected to foot shock (emotional stress, EmS); P67–P68: electrocardiogram (ECG) before “double stress”; P71–P72: ECG after “double stress”; black rectangle: decapitation and collection of biomaterial.

After mating, pregnant females were kept in individual plastic cages (36 × 20 × 14 cm) until delivery (postnatal day 0, P0). On P1, the rat pups were separated from their mothers and weighed, their sex was determined, and then the pups were re-distributed to nursing females. For reducing the influence of the genetic factor and the maternal care factor, 6–8 male and female rat pups from different litters born on the same day were left with each dam. These mixed litters were randomly assigned to two groups according to inclusion/exclusion criteria. The groups included male and female Wistar rats from at least six different litters, born on the same day, somatically healthy. Sick animals and some pups from very large litters were excluded so that all lactating females had no more than 6–8 pups. The sample size was determined based on articles published by other authors with a similar study design. One group consisted of the pups that were subjected to the procedure of daily separation from their mother and isolation from siblings for 6 h on P2–P18 (group “Maternal Separation and Isolation”, MSI). The boxes (6 × 6 × 5 cm) with separated rat pups were placed in a special room with a temperature of 22–23 °C. The other group included pups that were handled and immediately returned to the female daily (Control group). The juvenile rats were weaned on PND24 and housed in new cages (57 × 37 × 19 cm). In each cage, animals of the same sex and nursed by one dam were housed. We performed two experimental series. In Series 1, approximately half of the randomly selected males and females were decapitated at the age of 28 days and brain tissue samples were obtained for monoamine assay. The remaining animals were not disturbed until the behavioral experiments at the age of 56–60 days, after which they were decapitated and the brain samples were obtained. In Series 2, after conducting a battery of behavioral tests at the age of 56–60 days, the animals were subjected to additional stress for 5 days for 10 min per day: some rats were subjected to unavoidable foot-shock stress (physical stress) and others witnessed this procedure (emotional stress). Before “double stress” and after it, electrocardiograms were recorded to assess the autonomic balance in rats (data under processing). Immediately before decapitation, the animals were placed in the context of the previous stress, but no electric shocks were inflicted. The scheme of the experiment is shown in Figure 1. The number of animals in groups is shown in Table 1 in the “Section 3”.

### 2.3. Behavioral Tests

The tests were carried out during daytime from 9.00 to 17.00; the cages with control and experimental animals were alternated (Control–MSI–Control–MSI, etc.) to reduce the influence of the daytime factor. Behavioral testing included assessment of motor (horizontal) and exploratory (vertical) activity in automated open field (aOF) and classical (cOF) tests. These tests differ in aversive conditions, in particular, by the size and shape of the open space and the intensity of illumination. The cOF is more stressful. It is known that testing in a large open space in a classical situation of OF testing aversive for animals allows for the detection of sex differences in animal reactions [47]. Anxiety-like behavior was evaluated by testing in cOF and an elevated plus maze (EPM). Rat social behavior was evaluated in a three-chamber social test (TST) and in a social interaction test (SIT). Each rat was tested only one time per day, and the interval between the tests was at least 24 h.

### 2.4. Automated Open Field

In the aOF test, an arena (48 × 48 × 21 cm) with transparent walls was used (OptoVarimex, Columbus Instruments, Columbus, OH, USA). The measurements were carried out in dimmed light (17 lux) for 10 min. Horizontal (motor) activity was evaluated by the traveled distance (in cm) and vertical (exploratory) activity was assessed by the number of rearings using the applied software. In this and all other tests, the experimental chamber was wiped with 70% alcohol and dried with a towel after each animal.

### 2.5. Classical Open Field

In the cOF test, we used a round white arena (diameter 120 cm, wall height 28 cm) divided into squares of 20 × 20 cm. Measurement was carried out for 5 min: 3 min in bright light (500–510 lux in the center, 400–410 lux near the wall), 1 min in red light (a 40 W lamp positioned at a height of 80 cm above the center of the OF), and 1 min again in bright light. Behavioral parameters were assessed visually. Motor activity was evaluated by the number of crossed squares, and exploratory activity was assessed by the number of rearing postures. Modulation of lighting conditions was used to evaluate the reaction to novelty, which was calculated as the ratio of the number of crossed squares during the fourth minute in red light to that during the first minute of the session in bright white light. In addition, the total activity over two minutes after switching off and returning to the bright light was taken into account.

### 2.6. Assessment of the Anxiety–Phobic Level

In cOF, the anxiety–phobic state of the rats was measured in bright light using several stress tests based on situations that could cause anxiety in rats: descent from a platform 10 cm high; exit from a transparent box (11 × 15 × 15 cm); passage through a hole (11 × 7 cm) separating two compartments of the chamber (25 × 23 × 23 cm); and exit from the center of the OF (for a detailed description see [48]). In each test, the behavior was scored from 0 to 3 based on the latency of the reaction. The total score served as the overall assessment of anxiety–phobic states for each animal.

### 2.7. Elevated plus Maze

The level of anxiety in the EPM was assessed using the equipment and software of the VideoMot2 video system (TSE System, Berlin, Germany). The maze was located 70 cm above the floor and consisted of two opposite closed arms (CAs) with side and end walls (43 × 14 × 22 cm), two open arms (OAs) of the same size but without walls, and a central platform (14 × 14 cm). The illumination was 29 lux in OA and 1 lux in CA. Rat behavior in the EPM was recorded using a standard approach [49] according to the modern protocol [50]. The rat was placed in the center of the maze with its head directed towards one of the ORs. The number of visits and the duration of stay in the OAs and CAs were assessed over 5 min, and the preference for ORs was calculated as the ratio of the time spent in OAs to the total duration of stay in arms of the EPM; the locomotor activity was assessed by the total distance traveled in the EPM (cm); the mean speed of movement (cm/s), the number and duration of vertical rearing postures, grooming, and hanging from the OR were also determined.

### 2.8. Social Interaction Test

In a SIT, social contacts of a rat with a conspecific are evaluated under the conditions of free behavior of both [51]. Testing was carried out in a plexiglass arena (57 × 37 × 19 cm) unfamiliar to the animals in red light (7 lux). We compared the behavior of rats in pairs that were formed in such a way that both animals were from either the experimental or the control group, did not interact with each other earlier, and had similar body weight (the difference did not exceed 15%). Before testing, the animals were kept in cages singly for 24 h to increase social activity. The duration of testing was 15 min. The duration of active non-aggressive social contacts (sniffing, social grooming—licking, crawling under or mounting a partner, and chasing without aggression) and aggressive contacts (chasing that turns into aggressive interaction, attacks/fights, bites, aggressive grooming—biting causing vocalization of the partner) were evaluated during testing.

### 2.9. Three-Chamber Social Test

The TST assessed the sociability of rats, i.e., the choice between an unfamiliar social object (a conspecific that was placed in a small cylinder cage restricting its motor activity) and a non-social object (an empty cylinder), as well as the preference for “social novelty” (the choice between familiar and unfamiliar conspecifics). The social testing apparatus was a gray plastic box 120 × 80 cm, divided with partitions into three equal chambers (40 × 80 cm). The partitions had openings allowing access to all chambers. Twenty-four hours before testing, each rat was placed in the empty three-chamber box with open doors and allowed to explore it for 5 min for adaptation to the experimental conditions. Intact rats of the same sex and age as animals of the experimental groups were adapted for 5 min to a cylinder cage (diameter 20 cm, height 30 cm) with metal wire walls. The intact rats were kept in a separate room of the vivarium during the study. The testing was performed in two stages.

In Stage 1, two cylinders, wire cages, were placed in two side chambers; the intact rat was placed in one cylinder as a social object, while the second cylinder was empty. The experimental rat was placed in the middle chamber with closed doors for 1 min for habituation, then the doors were opened and the rat could explore all three chambers for 10 min, after which the doors were closed again, and the experimental rat was returned to the middle chamber. In Stage 2, another intact rat (a new, unfamiliar social object) was placed into the empty cylinder. Then the doors were opened, and the experimental rat was allowed to explore all three chambers for 10 min. The parameters recorded at each stage were the time spent by the rat in each chamber with the objects, the number of visits to these chambers, the time spent near each object, and the number of approaches to the objects (sniffing the rat in the wire cage or the empty wire cage, i.e., active interest). Sociability in Stage 1 was assessed by the preference for a social chamber and a social object by the following formulas:

[(time spent in the chamber with the social object) − (time spent in the chamber with the empty cylinder)]/(total time spent in these two chambers) × 100%;

[(time spent near the social object) − (time spent near the empty cylinder)]/(total time spent near these two objects) × 100% [52].

In Stage 2, the preference for social novelty was assessed by the preference for a chamber with the novel social object (unfamiliar rat) and the new social object itself vs. familiar social object. The animals that did not explore all three chambers during the habituation session and the animals that did not visit one of the side chambers during stage 1 were excluded from the study.

### 2.10. Additional Repeated Stress Exposure of Adult Rats

Patients with BPD may differ from healthy individuals in the functioning of the stress response system, and the most traumatic situation for them is psycho-emotional stress during social interaction [53,54]. For modeling psycho-emotional stress, the rats were divided into pairs: each pair consisted of rats of the same group (Control or MSI) familiar with each other. The rats were placed in a chamber divided into two compartments (25 × 23 × 23 cm) by a transparent partition with holes. One animal from a pair was subjected to unavoidable weak electric foot shocks for 5 consecutive days (Foot Shock subgroup, FS): the session lasted 10 min and consisted of 10 shocks (0.8 mA, 5 s) delivered at pseudorandom intervals [55]. The second animal witnessed this procedure and received olfactory, auditory, and visual signals from the rat subjected to electrical stimulation (Emotional Shock subgroup, EmS). After the last stimulation procedure, the reactivity of the autonomic nervous system of the rats was assessed using a cardiorhythmogram (see Figure 1). One day after ECG recording, before decapitation, the animals in the same pairs were placed in the chamber for 5 min and the corresponding rat received one shock on the paws.

### 2.11. Measurement of the Level of Monoamines and Their Metabolites in Brain Structures

After decapitation, the brain was removed, placed on a cold surface, and the frontal cortex, hippocampus, striatum, and hypothalamus were isolated according to Paxinos and Watson [56] and Chui et al. [57]. The tissue samples were frozen in liquid nitrogen, weighed, and stored at −80 °C.

In the brain tissue samples, the concentrations of biogenic amines norepinephrine (NE), dopamine (DA), 5-hydroxytryptamine (5-HT), and their metabolites 3,4-dihydroxyphenylacetic acid (DOPAC) and homovanillic acid (DA metabolites) and 5-hydroxyindoleacetic acid (5-HIAA; 5-HT metabolite) were measured using High-Performance Liquid Chromatography with Electrochemical Detection (HPLC-ED). The samples were homogenized with a Labsonic M ultrasonic homogenizer (Sartorius, Aubagne, France) in 0.1 n HClO4 (Sigma Aldrich, St. Louis, MO, USA) with 250 pmol/mL internal standard 3,4-dihydroxybenzylamine hydrobromide (Sigma Aldrich, St. Louis, MO, USA). After that, the samples were centrifuged for 15 min at 2000× *g*.

HPLC separation was carried out on a reversed-phase column ReproSil-Pur, ODS-3, 4 × 100 mm with a pore diameter of 3 µm (Dr. Majsch, Ammerbuch, Germany) at 26 °C and a mobile phase speed of 1 mL/min coupled with an LC-20ADsp liquid chromatograph (Shimadzu, Kyoto, Japan). The mobile phase consisted of 0.1 M citrate–phosphate buffer pH 2.58, 0.3 mM sodium octanesulfonate, 0.1 mM EDTA, and 8% acetonitrile (all reagents from Sigma Aldrich, St. Louis, MO, USA). The electrochemical detector Decade II (Antec Leyden, Zoeterwoude, The Netherlands) was equipped with a working glass carbon electrode (+0.80 V) and an Ag/AgCl reference electrode. Peaks of interest and the internal standard were identified by their elution time in the standard solution. The concentrations of amines were calculated by the internal standard method using a calibration curve using LabSolutions software (Shimadzu, Japan). The samples were normalized to tissue weight. In addition, the 5HIAA/5HT, DOPAC/DA, and HVA/DA ratios were calculated.

### 2.12. Statistical Processing of the Results

The data were processed using Statistica for Windows 7.0 software. If the hypothesis on normal data distribution was not rejected after preliminary testing using the Kolmogorov–Smirnov test, parametric methods were used. The effect of factors Sex (2 gradations—male or female), Rearing (2 gradations—growing with mother (Control) or maternal separation and isolation from littermates (MSI)), and Stress (2 gradations: physical stress (FS) and emotional stress (EmS)) were assessed using a multivariate ANOVA. The intergroup differences were evaluated using a Newman–Keuls post hoc analysis. If the hypothesis on normal data distribution was rejected, the nonparametric two-way Mann–Whitney U test for independent variables was applied with adjustment for multiple comparisons using the FDR method [58]. The significance level was set at 5%. The data are presented as M ± S.E.M (in the case of normal data distribution) or as the median and the first and third quartiles (when the data distribution did not correspond to the normal law).

## 3. Results

### 3.1. Body Weight

In Series 1, factors *Sex* and *Rearing* did not affect the body weight of the animals at the age of 1 and 2 months (Table 1). In Series 2, the body weight of animals was influenced by the *Sex* factor at the age of 2 (P58) and 3 (P75) months: males had greater body weight than females, but no differences between 2-month-old females and males within the groups (Control and MSI) were found (see Table 1). The influence of the factor of Rearing reached the level of statistical significance at the age of 3 months. The rats subjected to maternal separation had lower body weight than controls.

### 3.2. Locomotor and Exploratory Activity

**Figure 2:**
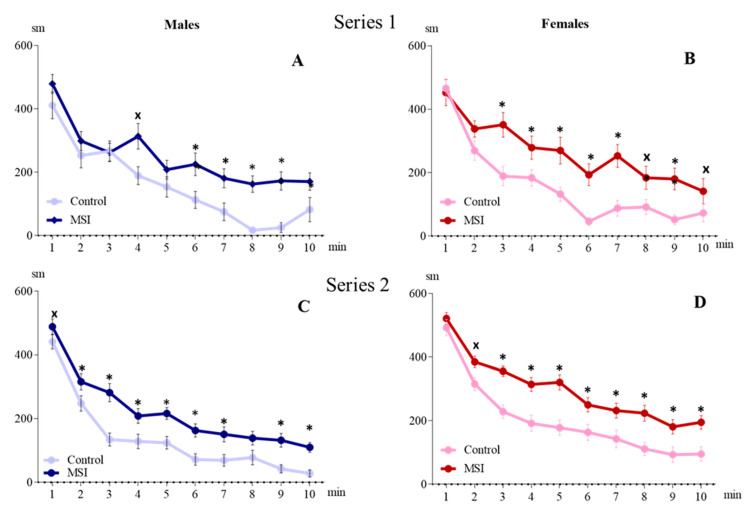
Locomotor activity in automated open field demonstrated by 2–month-old rats subjected to early-life maternal separation stress (MSI, dark lines) and normally reared rats (Control, light lines): (**A**,**C**)—males (Series 1 and 2, respectively); (**B**,**D**)—females (Series 1 and 2); * *p* < 0.05; ^x^ *p* < 0.07 in comparison with the Control (Newman–Keuls test).

In both series, the influence of the factor of Rearing on the total (over 10 min) locomotor activity of rats in aOF was revealed: in Series 1 F (1, 49) = 25.318, *p* < 0.001; in Series 2 F (1, 92) = 50.053, *p* < 0.001. In MSI groups, locomotor activity in both males (Figure 2A,C) and females (Figure 2B,D) was higher than in the Control group. In Series 2, locomotor activity was influenced by the factor of Sex (F (1, 92) = 26.460, *p* < 0.00): in females, the distance traveled was longer than in males in both the Control group (2014.08 ± 145.39 and 1366.38 ± 113.55 cm, *p* < 0.001) and the MSI group (2980.59 ± 129.06 and 2269.17 ± 139.39 cm, *p* < 0.001).

**Figure 3:**
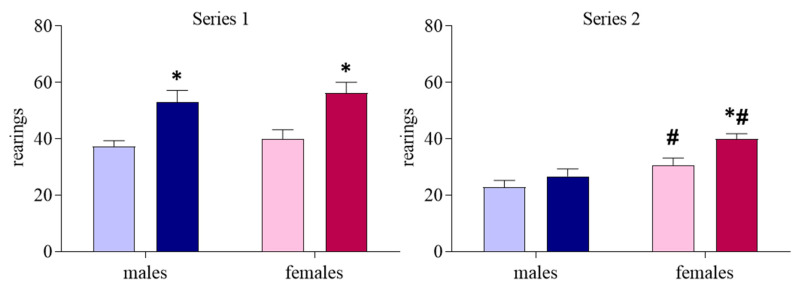
Exploratory activity in automated open field demonstrated by 2–month-old rats subjected to early-life maternal separation stress (MSI, dark bars) and normally reared rats (Control, light bars). *: *p* < 0.01 in comparison with control; #: *p* < 0.01 in comparison with males of the corresponding group (Newman–Keuls test).

In Series 1, males and females of the MSI groups demonstrated higher exploratory activity than animals of the corresponding Control groups (Rearing main effect: F(1, 49) = 17.073, *p* < 0.001) (Figure 3). In Series 2, a statistically significant increase in exploratory activity in MSI groups relative to the Control was observed only in females (Rearing main effect: F(1, 92) = 9.635, *p* = 0.003). In both Control and MSI groups, females had higher exploratory activity than males (Sex main effect: F (1, 92) = 18.945, *p* < 0.001).

**Figure 4:**
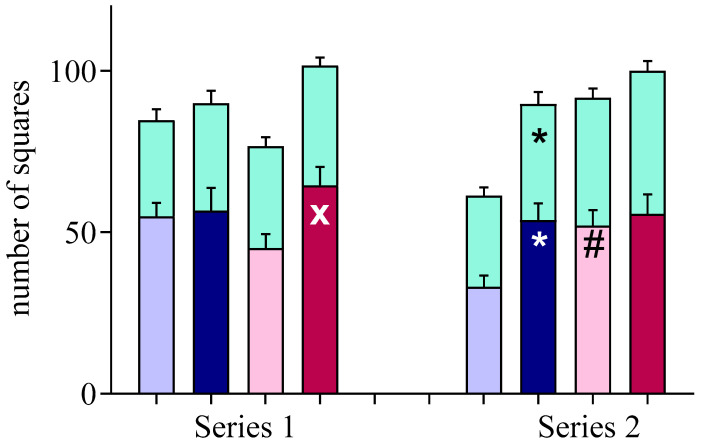
Locomotor activity over 3 min in classic open field demonstrated by 2-month-old rats subjected to early-life maternal separation stress (MSI, dark bars) and normally reared rats (Control, light bars). Green bars: locomotor activity under conditions of alternating lighting conditions (4th and 5th minutes of the test). *: *p* < 0.01 and x: *p* < 0.08 in comparison with the Control; #: *p* < 0.01 in comparison with males of the corresponding group (Newman–Keuls test).

In the cOF test under bright illumination (1–3 min), the level of locomotor activity was influenced by the factor of Rearing: motor activity in the MSI group was higher than in the Control group in Series 1 (F (1, 47) = 4.325, *p* = 0.043) and Series 2 (F (1, 92) = 6.101, *p* = 0.015). Note that in Series 1, an increase in motor activity was observed in female MCI (pronounced trend), and in Series 2, in male MCI (Figure 4). The influence of the factor of Sex was observed only in Series 2 (F (1, 92) = 4.543, *p* = 0.036): in females, activity was higher than in males. Under conditions of changing illumination (fourth and fifth minutes of the test), the influence of the factors Rearing and Sex was revealed in Series 2 only: (F (1, 92) = 4.775, *p* = 0.031 and F (1, 92) = 11.061, *p* = 0.001, respectively). In Series 1, the Rearing (F (1, 47) = 2.405, *p* = 0.128) and Sex (F (1, 47) = 0.040, *p* = 0.842) factors did not affect motor activity. The level of exploratory activity of the rats did not differ significantly in all subgroups.

### 3.3. Anxiety-like Behavior

**Figure 5:**
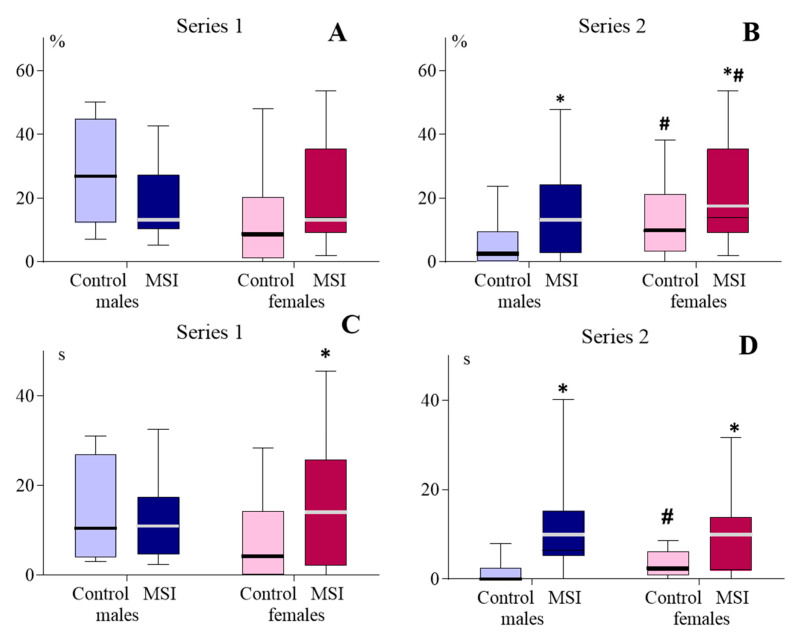
Anxiety level in EPM demonstrated by 2-month-old rats subjected to maternal separation stress in early life (MSI, dark bars) and normally reared rats (Control, light bars). (**A**,**B**)—preference for open arms; (**C**,**D**)—duration of hanging from open arms; * *p* < 0.01 in comparison with the control; # *p* < 0.01 in comparison with males of the corresponding group (Mann–Whitney U test).

In the EPM, the influence of the interaction of the factor Rearing × factor Sex on the total locomotor activity was revealed in Series 1, (F (1, 49) = 4.672, *p* = 0.030): in MSI females, the total distance traveled in EPM was greater than in the Control (1694.98 ± 158.47 and 2217.11 ± 150.06 cm, respectively; *p* < 0.05, Newman–Keuls test). Only MSI females showed an increase in the duration of hanging from the OA in comparison with the Control (Figure 5C). The differences between the MSI and Control groups in the preference for OA in Series 1 did not reach the level of statistical significance (Figure 5A).

In Series 2, the Rearing main effect F (1, 92) = 6.688, *p* = 0.017 was statistically significant for locomotor activity in OAs: the distance traveled in OAs increased in both males (229.37 ± 38.31 cm in MSI vs. 103.72 ± 25.77 in the Control; *p*< 0.05, Newman–Keuls test) and females (340.60 ± 45.30 cm in MSI vs. 209.87 ± 36.97 in the Control; *p* < 0.05). For total locomotor activity, the Rearing main effect was F (1, 92) = 12.368, *p* < 0.001 and Sex main effect was F (1, 92) = 7.010, *p* = 0.009. MSI males accumulated longer distances in the EPM than Control males (2026.71 ± 77.49 and 1427.83 ± 134.63, respectively, *p* < 0.01). In females, the differences did not reach the level of statistical significance (Control—1976.82 ± 86.57; MSI—2064.44 ± 88.34), The preference for OAs (Figure 5B) and duration of overhangs from OAs in MSI animals of both sexes (Figure 5D) was greater than in the corresponding Control. The number of overhangs from OAs in MSI exceeded the control values: for male Control—0.0 [0.0; 2.0], for male MSI—6.0 [0.0; 9.0], *p* < 0.001; for female Control—3.5 [1.0; 5.0], and for female MSI –7.5 [0.0; 10.0]. *p* = 0.013, Mann–Whitney U Test).

In cOF, no differences in the indicators of the anxiety–phobic level scale were found in both series.

### 3.4. Social Behavior

**Figure 6:**
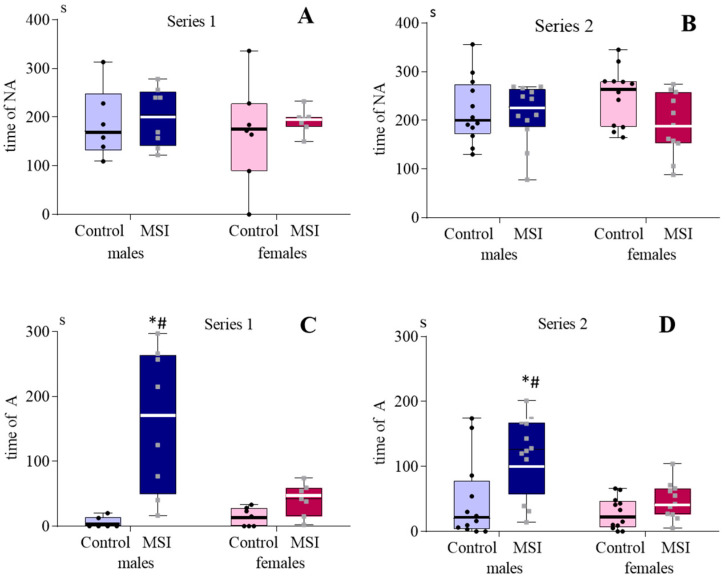
Time of non-aggressive (**A**,**B**) and aggressive (**C**,**D**) social contacts in 2-month-old rats subjected to early maternal separation stress (MSI) and normally reared rats (Control). *: *p* < 0.01 in comparison with the control; #: *p* < 0.01 in comparison with the corresponding group of females, *p* = 0.029 in Series 1 and *p* = 0.006 in Series 2 (Mann–Whitney test). Histograms for the number of aggressive and non-aggressive contacts were similar.

In SIT, MSI males and females did not differ by the number and duration of non-aggressive social contacts from the corresponding Controls in Series 1 and 2 (Figure 6A,B). In both series, the number and duration of aggressive interactions in the MSI surpassed the corresponding parameters in Control, but only in males were the differences significant (Figure 6C,D).

**Figure 7:**
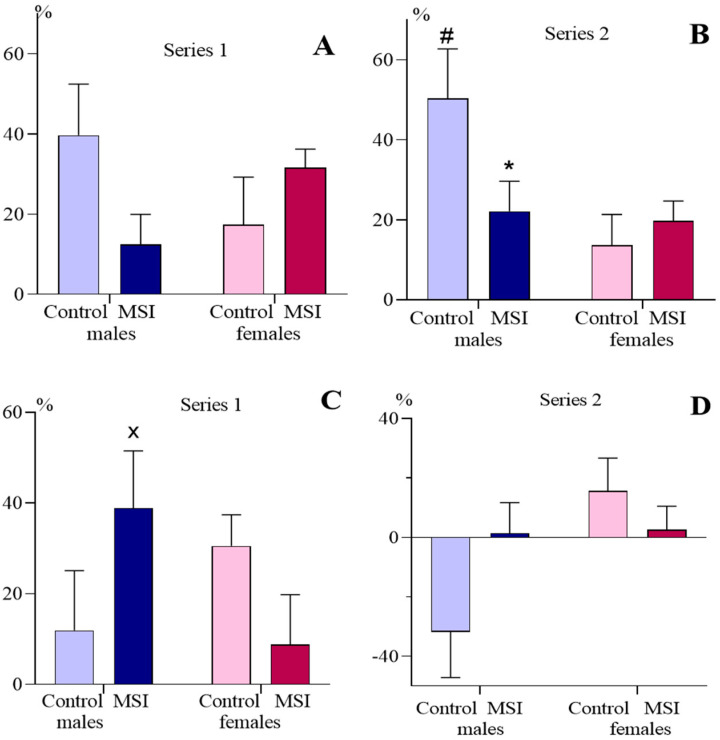
Preference for the social chamber (**A**,**B**) and for the chamber with an unfamiliar rat (**C**,**D**) in 2-month-old rats subjected to early maternal separation stress (MSI) and normally reared animals (Control).* *p* < 0.01 and x *p* < 0.08 in comparison with the Control; # *p* < 0.01 in comparison with the corresponding group of females (Newman–Keuls test).

In the TST in both series, we revealed interaction of the factor Rearing × factor Sex for the preference for the social chamber and preference for the chamber with an unfamiliar rat: for Series 1 F (1, 48) = 4.681, *p* = 0.035 and F (1, 46) = 4.587, *p* = 0.037, respectively; for Series 2 F (1, 79) = 4.303, *p* = 0.041 and F (1, 79) = 2.784, *p* = 0.099 (trend), respectively. Only in MSI males was the preference for the social chamber reduced in comparison with the corresponding values in control males; in Series 1, the intergroup differences did not reach the level of statistical significance (Figure 7A,B). A tendency to higher preference for the chamber with a new social object was revealed in MSI males in comparison with the Control (Figure 7C,D). In Series 2, the preference for a social chamber was influenced by factor Sex (F (1, 79) = 5.669, *p* = 0.208: Control males demonstrated higher preference for a social chamber than Control females, while in the MSI group, no differences in this parameter were revealed between males and females (see Figure 7B).

### 3.5. Monoamines

#### 3.5.1. Norepinephrine

At the age of 1 month after weaning, the Rearing main effect was significant for the hippocampus, and the Sex main effect and Sex × Rearing interaction were significant for the hypothalamus (Table 2). Post hoc analysis revealed an increase in the level of NE in these structures only in MSI males compared with the Control.

At the age of 2 months after a battery of behavioral tests, as well as at the age of 1 month, the Rearing main effect was significant for the hippocampus, and Sex × Rearing interaction was significant for the hypothalamus. However, the NE level was reduced in the MSI group compared with the Control only in the hippocampus of males and in the hypothalamus of females. In the frontal cortex and striatum, no differences in NE levels were revealed at the ages of 1 and 2 months.

In adult animals after repeated stress, the Stress main effect was significant for the frontal cortex and for the hippocampus (see Table 2). After the EmS, the level of NE was reduced in the frontal cortex in MSI females and in the hippocampus in MSI males compared with the corresponding Control.

#### 3.5.2. Dopamine

At the age of 1 month, **in the frontal cortex,** the Rearing main effect was significant for HVA level and HVA/DA ratio: these parameters in the MSI were lower than in the Control (Table 3). Post hoc analysis showed a statistically significant decrease in HVA level and DA turnover in MSI females. **In the striatum,** the influence of Sex × Rearing interaction was revealed for DOPAC and HVA: their levels were reduced in MSI males compared to Control. The Sex main effect was significant for DOPAC levels and for DOPAC/DA and HVA/DA ratios: all these parameters were less in females than in males. **In the hypothalamus,** Sex × Rearing interaction influenced the levels of DA, DOPAC, HVC, and DOPAC/DA ratio. The level of DA was reduced in MSI males in comparison with Control; the DOPAC/DA turnover was increased in MSI females; and the HVA/DA turnover was elevated in the total MSI group. The levels of DOPAC and HVC demonstrated opposite changes: they decreased in males and increased in females, but these shifts did not reach the level of statistical significance in a posteriori analysis. **In the hippocampus**, no differences in the analyzed indicators between the subgroups were found.

At the age of 2 months, **in the frontal cortex and hippocampus,** only the main effect of Sex was shown. The HVA level was reduced in females in the frontal cortex. The DA turnover (DOPAC/DA ratio) in females in the frontal cortex was lower, and in the hippocampus, it was higher than in males. **In the striatum,** the Rearing main effect was significant for the levels of DA, HVA, and the DOPAC/DA and HVA/DA ratios, while the Sex main effect was significant for the levels of DA and HVA. The DA level was higher in females than in males and decreased in MSI females compared to Control. The DOPAC/DA ratio, on the contrary, was lower in females than in males and increased in MSI females compared to the Control. In MSI males, the level of HCV and HVC/DA turnover were reduced in comparison with the Control. **In the hypothalamus,** the Rearing main effect was revealed for the levels of DOPAC and HVA and the ratios of DOPAC/DA and HVA/DA. The DOPAC level in MSI rats was lower than in the Control; no differences between the subgroups were found. The DOPAC/DA ratio was elevated in Control females in comparison with the males; in MSI females, the DA turnover was decreased compared to the Control. The HVC level and the HVA/DA ratio were higher in females than in males and reduced in both males and females of the MSI group.

After repeated stress, **in the frontal cortex**, the DOPAC/DA ratio was lower under EmS conditions than under FS conditions as a result of the Stress factor. **In the striatum**, the Stress main effect was significant for the DA level and its metabolites, DOPAC and HVC, which were higher after EmS than after FS. **In the hypothalamus**, DA turnover (DOPAC/DA) was significantly reduced under conditions of EmS in comparison with FS.

#### 3.5.3. 5-Hydroxytryptamine

At the age of 1 month, **in the hippocampus,** the influence of the factor Rearing on the level of 5-HT was revealed: 5-HT level was elevated in MSI rats (to a greater extent in males, *p* = 0.06, Newman–Keuls test) (Table 4). **In the striatum**, we revealed the influence of the factor Sex on the level of 5-HT (in females it was higher than in males in both the experimental and control groups) and on the 5-HT/HIAA ratio (this parameter in females was lower than in males). **In the hypothalamus,** the Rearing main effect was significant for the 5-HT level, while the Sex × Rearing interaction was significant for the HIAA level. Both indicators were reduced in MSI males compared to the Control. **In the frontal cortex,** no differences between the groups were revealed.

At the age of 2 months, **in the frontal cortex**, a Sex × Rearing interaction was revealed for the levels of 5-HT and HIAA: they were increased in males and reduced in females in comparison with the corresponding Control subgroups; for the HIAA/5-HT ratio, the Sex main effect was significant: the turnover of 5-HT was lower in females than in males. **In the hippocampus**, the Sex main effect was revealed for the levels of 5-HT (lower in females than males) and for HIAA and the HIAA/5-HT ratio (higher in females than in males); no differences between the subgroups were found. **In the striatum**, a Sex main effect was identified: the 5-HT level and HIAA/5-HT ratio in females were lower than in males. The Rearing main effect was significant for HIAA level: it was higher in MSI rats. **In the hypothalamus**, the levels of 5-HT and HIAA were lower in MSI males, and the HIAA/5-HT ratio was lower in MSI females than in the corresponding Control.

Under conditions of repeated stress in adult rats, the influence of Sex × Stress interaction on the HIAA level **in the hippocampus and hypothalamus** was revealed: in males, the level of this metabolite in animals subjected to EmS was higher than in animals subjected to FS.

## 4. Discussion

Repeated maternal separation is the most widely used animal model for investigating the relationship between early life stress and its psychiatric and physical consequences. Despite non-standardized protocols, the MS paradigm is mainly considered as a tool for modeling anxiety- and depression-like behavior of juvenile and adult rodents [38,42,59,60,61]. Most commonly, the pups are separated from their mother for 3–6 h each day, beginning on P1–P2 and continuing through either P14–P21. Other aspects of the protocol (isolation from one another, warmed or not warmed, light or dark cycle, handled or not handled control animals) are less consistent across studies.

In our experiments, complex developmental trauma was used, including separation of rat pups from the mother and littermates at room temperature with partial limitation of motor activity due to the small size of the boxes. We expected that early-life abuse would lead to attachment disorder in rat pups and to formation in later life of persistent neurobehavioral deficits not associated with the development of depression-like phenotype. It was shown that more pronounced disorders in behavior and neurotransmission were observed in rats subjected to complex aversive influences during brain development [62,63].

In two series of experiments, maternal separation and sibling isolation in the neonatal period led to similar changes in the behavior of young adult animals. In Series 1, we evaluated the effect of early-life stress on the levels of monoamines and their metabolites in the frontal cortex, hippocampus, striatum, and hypothalamus of juvenile and adult animals (Table 5). These brain structures and neurochemical systems were chosen because they have close functional relationships with changes in the emotional behavioral profile induced by early-life maternal and sibling separation stress. In Series 2, adult animals with behavioral disorders caused by early stress were exposed to repeated physical or psycho-emotional stress. The levels of neurotransmitters and their metabolites in the main projection areas of the monoaminergic systems was also assessed in these rats.

### 4.1. Body Weight

Body weight is one of the basic indicators of physical status in animals. Early life experiences have a strong influence on brain programming and can affect the control of eating behavior and body weight later in life. However, there is no consensus about the relationship between neonatal stress and eating behavior. Many researchers reported that rats subjected to early-life maternal separation had lower body weight in the juvenile and mature age [38,59,60,61,62,63,64,65,66,67], while reports on increased body weight are rare [68]. In our study, the body weight in adult animals subjected to MSI was below the control values. Moreover, in males, a tendency to weight loss in the stressed group was observed in all periods of observation, but only in females was body weight loss statistically significant, at the age of 3 months. It is considered unlikely that the weight loss was due to poor nutrition caused by MSI, because the nutritional status of rat pups (total protein, albumins, etc.) was not impaired [64]. Deficiency in physical development in MSI rats was primarily due to reduced food intake in later terms, which, in turn, is associated with hormonal shifts (changes in the levels of leptin and ghrelin [65], corticosterone [64]) and/or orexigenic peptides of the hypothalamus [69]). There is evidence that the 5-HT system is involved in the modulation of eating behavior: 5-HT transmits signals in the arcuate nucleus of the hypothalamus through the 5-HT1a and 5-HT2b receptors and increases food intake by expressing Creb and subsequent activation of several genes affecting appetite [70]. Leptin reduces appetite, at least partially, by inhibiting the synthesis of 5-HT in the brain stem. We observed a decrease in 5-HT level in the hypothalamus in juvenile and adult rats subjected to MSI, which can indirectly confirm the hypothesis of Yadav and colleagues [70]. It should be noted that this decrease was statistically significant only in males (Table 5), which can be explained by sex-related differences in food intake regulation by 5-HT. Our observations are similar to the results obtained by de Lima et al. [71]: maternal separation led to a decrease in 5-HT level in the hypothalamus of male and female rats, and males were more vulnerable than females to the effects of neonatal stress on food intake and changes in the 5-HT level in brain structures. On the other hand, Oh et al. [63] reported that complex stress with additional immobilization of rat pups after separation from the mother leads to a greater decrease in body weight in females than in males; the functional activity of the 5-HT system was also reduced in females. Perhaps females are more sensitive to complex trauma. However, the neurochemical mechanisms of the effect of complex trauma on feeding behavior (in particular, the role of 5-HT in appetite regulation) in males and females remains unclear and requires further study [72].

### 4.2. Locomotor Activity, Exploratory Activity, and Anxiety-like Behavior

In both series of our study, adult male and female rats subjected to repeated early MSI showed increased locomotor and exploratory activity in OF in comparison with the Control. The MSI-exposed rats demonstrated increased exploratory activity in aOF in dim lighting conditions. Under the bright light in the cOF, the vertical activity in the MSI groups did not differ from the control, which may be due to a decrease in the exploratory behavior of these rats under stressful conditions. We observed a decrease in signs of anxiety in MSI females in EPM: they had an increased preference for open arms and number and duration of hangings from the open arms. In males, the effect of early stress on anxiety-like behavior was less certain and was reduced only in Series 2.

The use of different early life stress schemes may have different effects on motor and exploratory activity and on the level of anxiety. So, maternal separation for 4 h on P2–P5, for 8 h on P6–P16, and weaning on P17 decreased locomotor activity, increased anxiety-like behavior and resulted in a passive coping strategy in Sprague–Dawley rats of both sexes [73]. Maternal separation for 3 h on P2–P14 did not lead to differences in total running distance in the OF test and anxiety-like signs between MS and control groups of Sprague–Dawley rats [74]. However, MS for 6 h during the first 2 weeks of life decreased orientation behavior and increased impulsivity in Sprague–Dawley juvenile males in an OF-test [39]. An anxiolytic effect of MS in 4–4.5-month-old male and female rats was detected in OF, but not in EPM [75]. The MS model similar to the procedure used in our study (P1–P15, 4 h, in individual boxes, in an incubator at 30 °C or at room temperature 22 °C) led to a significant increase in locomotion and exploratory behavior at the age of 2 months in male Wistar rats [76]. The hyperlocomotor phenotype was more pronounced in animals that had previously been isolated at room temperature. These rats also demonstrated more risk assessments, which is interpreted as an increase in the level of exploratory activity, and a decrease in anxiety behavior. The differences in the results can be explained by the use of animals of different lines, the peculiarities of MS protocols, the conditions of behavioral tests, and different ages of animals at the time of behavioral testing. Thus, it was shown that maternal separation (3 h, P2–P14) causes an increase in motor and exploratory activity in Wistar rats, but not in Wistar–Kyoto rats [77]. It is hypothesized that Wistar rats have more flexible survival strategies than WKY rats. This, however, does not necessarily mean that their response is more adaptive [78], because these strains exhibit similar drug abuse susceptibility.

The results obtained in this block of behavioral tests indicate that the protocol of neonatal complex aversive exposure applied by us leads to the formation of a hyperactive phenotype in young adult rats. The other authors have shown that a complex aversive effect (social isolation from P14 after 2-week MS) interferes with the normal process of cell proliferation, apoptosis, and synaptogenesis in the frontal cortex [40] and hippocampus [79] and leads to the formation of a hyperactive behavioral profile in juvenile and adult rats.

### 4.3. Social Behavior

In our study, sex differences in the effects of MSI on the social behavior of animals were revealed. The MSI males demonstrated increased aggressiveness under conditions of free interaction of animals in an unfamiliar environment, reduced preference for socializing, and increased reaction to the presence of social novelty under the conditions of restricted motor activity of conspecifics.

A number of studies have demonstrated that MS disrupts the social behavior of rats at a later age, and males are more sensitive to early-life stress. In juvenile Wistar males, daily long-term MS caused an increase in the number of aggressive elements in social play behavior in a social interaction test [80] and more attacks than normally reared males in the resident–intruder paradigm [81]. These animals had higher plasma levels of corticosterone and higher expression of vasopressin mRNA in the paraventricular nucleus of the hypothalamus. In adult Wistar rats, intermale aggression in this test and arginine–vasopressin immunoreactivity in the hypothalamus were also significantly higher in the MS group [82]. At the same time, immunoreactivity of 5-HT in the anterior hypothalamus was reduced and negatively correlated with the level of aggression. Our data show a decrease in the level of 5-HT and functional activity of the 5-HT system in the hypothalamus in juvenile and adult rats of the MS group, with this effect more pronounced in males. It should be noted that the content of 5-HT and HIAA in the frontal cortex of male MS rats was increased in comparison with the control. The forebrain cortex is involved in the formation of aggressive motivation in rats [83]. Impulsive social aggression is associated with a 5-HT deficit in the medial prefrontal cortex and a decrease in the inhibitory control of aggressive reactions [84,85]. However, the above studies analyzed reactive changes in the 5-HT level in the cortex at the moment of aggression. At the same time, the basal level did not differ in control and experimental (rats denied maternal reward in early life) animal groups.

We revealed no changes in sociality (sociability) in rats of both sexes by the results of SIT: the number and duration of non-aggressive interaction did not differ in MS and control groups. These results are at odds with the conclusions of Mintz et al. [86] that MS at room temperature led to a decrease in social motivation in adult male Wistar rats. This contradiction can result from the method of assessing social interaction: Mintz and colleagues used a larger arena (146 × 146 cm) for OF testing and estimated the time spent by the animals in a certain square of the field (home base) together or alone (social or solitary occupation) over three 10 min intervals. The rats subjected to MS at low temperature demonstrated decreased sociability only in the last third of the session. In our work, we evaluated only direct tactile contacts of the animals in a small arena (57 × 37 cm) over 15 min. In this time interval, Mintz and colleagues [86] also did not observe differences in sociality between the MS rats and controls.

Various studies of the effect of MS on social preference and social novelty in adult rats have yielded contradictory results. There is data that the social behavior of adult MS rats does not change [87]. Other data show that MS led to a sex-dependent dysfunction in the social interaction of rats. It is reported that MS males showed decreased social interaction with unfamiliar conspecifics without affecting their sociality, which, according to the authors, indicates a decrease in the flexibility of social behavior [88,89]. Zeng et al. [42] note that after postnatal stress, females developed social avoidance, while males began to interact for longer times with the partner rat; the rats of the control group preferred non-social objects.

The design of our experiment (animal strain, MS protocol, procedure of assessing social preference and social novelty) was most close to that of the study of Kambali and colleagues [89]. However, our findings partially differ from the results of this study. The identified changes in social behavior may be associated with impaired social motivation and/or social recognition in males exposed to MSI. Our data do not currently allow us to make a correct conclusion about the specificity of effects of MSI on animals of different sexes and to discuss the mechanisms underlying social behavioral deficits. This direction needs further research.

### 4.4. Monoamines

The aversive events during the critical periods of the development of an organism can disrupt the maturation of many neurotransmitter systems, including monoaminergic ones (see review [90]). The long-lasting impairment in the functional activity of these systems can be reflected in the levels of mediators and their metabolites in target areas of the brain. In our study, MSI rats showed changes in the levels of NE, DA, and 5-HT and their metabolites at the ages of 1 and 2 months. Sex- and region-specific features in the functional activity of monoaminergic systems caused by early life stress were revealed.

In 1-month-old males, the NE level was increased in the hippocampus and hypothalamus; the DA level was decreased in the hypothalamus, and DOPAC and HVC levels were decreased in the striatum; the 5-HIAA level was decreased in the hypothalamus (Table 5). In females, a statistically significant decrease in the level of HVA and the ratio of HVA/DA were observed in the frontal cortex, while in the hypothalamus, DA turnover, on the contrary, was increased and 5-HT level was reduced. In 2-month-old males, the level of NE decreased in the hippocampus, in contrast to that observed in 1-month-old males. Interestingly, in females, NE levels decreased in the hypothalamus. The shifts in the activity of the DA system were unidirectional, with changes in juvenile MSI rats. In the striatum, the HVA level and HVA/DA ratio decreased in males while the DA level decreased but the DOPAC/DA ratio increased in females. In the hypothalamus, the levels of DA metabolites and turnover of DA were decreased in both sexes. In the frontal cortex, there were no changes in the DA system, but the levels of 5-HT and 5-HIAA changed oppositely in animals of different sexes: they increased in males and decreased in females. Regardless of gender, activity of the 5-HT system decreased in the hypothalamus.

Since behavioral tests were not performed after weaning at 1 month of age, we are unable to assess the relationship between changes in monoamine levels and behavioral disturbances in adolescent rats. However, according to the literature, the dysfunction of NE and DA neurotransmission in animals is often associated with the development of a hyperactive phenotype [90]. The increased level of NE is usually associated with an increase in anxiety-like behavior and impulsivity. According to published reports, in the brains of Wistar rats that were subjected to MS (P0–P6) in combination with additional stressors (low temperature, bright light, and noise), increased levels of NE and DA were revealed immediately after the completion of the stress procedures (P7); on P20, the level of neurotransmitters decreased, but still remained above the control values [91]. In another study [92], after intermittent MS for 6–12 h in the first two postnatal weeks, the NE content was increased in the cortex and hypothalamus of rats on P21, and the DA content, on the contrary, was reduced. By P60, the level of NE returned to normal in all structures, while the DA level did not recover. Several lines of evidence point to an intricate reciprocal relationship between NE and DA in terminal areas, and their interaction, apparently, is involved in disrupting animal sociability [93]. At the behavioral level, juvenile and adult rats demonstrated increased exploratory activity and emotional tension [94]. However, there is evidence that MS leads to pronounced dysregulation in the neural connections of the midbrain DA regions in juvenile males; that being said, only adult rats demonstrated anxiolytic behavior [95].

In adult rats, we observed motor and exploratory hyperactivity in both sexes, increased aggressiveness and decreased sociability in males, and signs of decreased anxiety in females. It is believed that all DA systems are involved in the development of the hyperactive phenotype [94,96]. According to this hypothesis, the hypofunction of the DA system plays a pivotal role in the development of behavioral deficits by failing to modulate nondopaminergic (primarily glutamate and GABA) signal transmission appropriately. In a recent review of animal models of affective and behavioral disorders (which include maternal deprivation), referred to as “reward deficit syndrome”, it is noted that these conditions are based on a low tone of the DA system [97]. Our data are consistent with this hypothesis, at least for the striatum and hypothalamus. It should be noted that at the age of 2 months, we did not reveal changes in the DA and metabolites levels in the frontal cortex, noted by many authors, although in adolescent rats, a decrease in the turnover of DA in the frontal cortex was noted.

Most researchers recognize the priority contribution of the DA system to the development of hyperactivity, but not everyone shares the point of view that the disorders are based on the hypofunction of all DA systems. Experimental data indicate that in some cases the symptoms of hyperactivity are associated with an increase in the functional activity of DA [90,93,98]. Two lines of rats with genetically determined hyperactivity (spontaneously hypertensive rats, SHR and Naples High Excitability, NHE) are similar in behavioral manifestations, but show different changes in DA systems [98]. Tyrosine hydroxylase is hyperexpressed in NHE rats and hypoexpressed in SHR. The DA transporter is hyperexpressed in both lines, although in the SHR, DAT activity is low (reduced DA uptake). The DA levels in the striatum and prefrontal cortex are increased in juvenile SHR, but are decreased in handled young and non-handled older animals. This contradiction can be explained by different degrees of involvement of cortical and subcortical mechanisms in the pathological process, which, in turn, is a result of interactions between individual predisposition and environmental factors.

It is well known that the 5-HT system plays an important role in the pathophysiology of anxiety, depression, and aggression [99]. Although the effect of maternal separation on the 5-HT system has been extensively studied, the results are contradictory among different labs. For instance, a single 24 h maternal deprivation increased 5-HT activity in the prefrontal cortex, hippocampus, and striatum of adolescent [100] and in the hypothalamus of adult Wistar rats [101]. Daily MS from P1 to P13 increased tissue levels of both 5-HT and 5-HIAA in the dorsal raphe nucleus and nucleus accumbens in female adult rats [102]. Other studies show that repeated MS procedures reduced basal levels of 5-HT in the dorsal hippocampus and medial prefrontal cortex of adult rats [45] and in the ventral striatum of rat pups [103]. Maternal separation at P1-P21 led to an U-shaped and inverted U-shaped patterns for 5-HT synthesis and 5-HT activity in the prefrontal cortex and nucleus accumbens from younger Wistar males to adults [104]. In our study, the level of 5-HT and its metabolite in the frontal cortex decreased in males and increased in females, which may be the neurochemical basis of differences in behavior.

### 4.5. Additional repeated Stress in Adult Rats

It has been proposed that early life negative events may alter the response of a subject to a subsequent traumatic experience, increasing the likelihood that later trauma will lead to PTSD or affective disorders [105]. Witnessing a traumatic event but not directly experiencing it can be psychologically quite damaging [106]. Some studies have reported the occurrence of emotional distress in animals that witness other animals suffering from foot shock [107]. Exposure to inescapable foot shock produces a series of behavioral consequences (“learned helplessness”) that may be prolonged by re-exposing organisms to the contextual cues associated with the original experience of inescapability, even if shock is not delivered during these “reminders” [108].

We expected MSI rats to be more sensitive to emotional impact than control animals. We evaluated the effect of prolonged (5 days) physical and emotional stress on the levels of monoamines and their metabolites in several areas of the brain in male and female rats reared with their mother or under conditions of MSI. Although the influence of the factors Sex, Rearing, and Stress as well as their interaction was identified, we did not obtain a wide range of differences between groups (see Table 2, Table 3 and Table 4). Statistically significant findings were elevated levels of NA in the frontal cortex in females of all groups compared with the values of indicators in the corresponding groups of males, which may indicate a greater sensitivity of females to stress. At the same time, in the MSI group under the conditions of EmS, the level of NA was lower than in Control in females in the frontal cortex and in males in the hippocampus. In addition, in the striatum of females, the level of DA was increased compared to males, and in all animals, the levels of DA and DOPAC were higher after EmS than after FS. In the frontal cortices of MSI rats of both sexes, DA turnover (DOPA/DA ratio) was reduced after EmS compared with FS. There were no intergroup differences in the functional activity of the 5-HT system under conditions of physical or emotional stress.

Early studies have demonstrated that physical and psycho-emotional stresses cause different regionally dependent changes in the level of DA [109] and 5-HT [110] in the brains of rats, and the effects of physical stress depend on the intensity of the stimulus. Psycho-emotional stress [109] or mild foot shock [111] selectively activate the mesocortical DA system and increase 5-HT metabolism only in the medial prefrontal cortex. On the contrary, severe physical stress stimulates DA and 5-HT metabolism in many brain structures (prefrontal cortex, nucleus accumbens, amygdala, and hypothalamus). According to the “double hit” hypothesis, early postnatal stress modulates the resilience/sensitivity of adult animals to stressful stimuli by modulating (among other mechanisms) activity of the brain neurotransmitter systems [30]. Recent studies have demonstrated that complex stress that included maternal separation in the early postnatal period, isolation during adolescence, and inescapable foot shock led to the appearance of more numerous and longer freezing episodes (tonic immobility) in the fear conditioning test in 2-month-old male rats in comparison with animals subjected to foot-shock stress alone and native controls [112]. This behavioral pattern can be associated with the inability of individuals exposed to early stress to integrate information and to respond appropriately to environmental stimuli. Behavioral disorders in adult animals can be a result of changes in the stress reactivity of the monoaminergic systems. Thus, immobilization stress in 2-month-old rats subjected to postnatal maternal deprivation caused anxiety-like behavior and a decrease in NE release in the hippocampus and hypothalamus and an increase in 5-HIAA levels in the frontal cortex and hippocampus [113]. In adult rats subjected to early prenatal stress, the activity of NE neurons of the locus coeruleus and DA neurons of the ventral tegmental area decreased after a forced swimming test, whereas in control animals, the activity of these neurons increased [114]. The authors interpreted these changes as adaptive, contributing to stress resilience.

## 5. Limitations

The first limitation of the current study is related to the determination of monoamine levels. The concentrations of monoamines and their metabolites were measured in relatively large areas of the brain and not in specific nuclei. Given the mediator and functional heterogeneity of the structures chosen for the study, the assessment of the contribution of monoaminergic systems to the development of behavioral disorders can be quite rough. More accurate bioassays of monoamines and their metabolites in these cores should be performed in future studies. In addition, we did not evaluate basal monoamine levels during an experiment in which animals were subjected to additional adult stress, so we cannot conclude the actual effect of physical or emotional stress on monoaminergic neurotransmission. Our data allow us to judge only the differences in response of monoaminergic systems to stressors of different natures. In the present study, we wanted to evaluate the effect of growing conditions on the level of monoamines and determined the content of neurotransmitters at the time of weaning. Therefore, we did not screen the behavior of adolescent animals, which makes it difficult to interpret the observed changes in the state of neurotransmitter systems of the brain. We conducted an additional series of studies in which the level of neurotransmitters in adolescents was determined after assessment of behavior. These results are being processed.

Another limitation is related to the complexity of assessing the influence of rat pup rearing factors on the formation of the endophenotype and behavioral profile of adult animals. Early stress, in particular maternal deprivation in rat pups, does not always lead to the development of post-traumatic stress disorder and other stress-related disorders in adulthood. There is no doubt that different outcomes of early trauma are associated with the plasticity of the endocrine, nervous, and immune systems. Plastic changes can be adaptive or maladaptive in nature and be mediated by environmentally dependent changes in active maternal care on the one hand and the rigidity of environmental influences on the other [115]. In our experiment, additional physical threats (low temperature and restrictions on locomotor activity during isolation of littermates) were aimed at inducing a sustained and reliable early life stress effect. However, in this case, we cannot separate the psychosocial effects (maltreatment or neglect) from the physical aversive effects. Addtionally, we did not assess maternal behavior during the MSI protocol to account for its effect on individual behavioral variability and vulnerability to stress in adult rats. These factors of environmental influences require additional control in subsequent experiments.

## 6. Conclusions

In the present study, we show that the protocol of maternal separation and isolation from siblings that we used led to the formation of a hyperactive phenotype in adult animals and disruption in emotional regulation and social interaction. At the same time, females were more characterized by a decrease in anxiety, while males were characterized by an increase in aggressiveness and a decrease in sociability.

Early life stress effects on brain monoamine concentrations were sex-dependent and region-specific. The opposite directions of 5-HT and 5-HIAA shifts in the frontal cortex may be associated with differences in behavior patterns in adult male and female rats.

An increase in the levels of DA and DOPAC in the striatum of animals of both sexes under conditions of witness stress may indicate an increased sensitivity to emotional stress in rats reared under conditions of deprivation of maternal care.

Thus, maternal deprivation in rodents, as a model of traumatic childhood experiences, is considered promising for studying neurodevelopmental disorders and subsequent vulnerability/resistance to the onset of mental disorders. Although our results show that MSI alters the levels of monoamines in brain structures associated with the development of stress-related diseases in adult rats, it remains unclear how these changes are recruited in the formation of endophenotypes and behavioral features of animals (including state/vulnerability to stress). Changes in the level of neurotransmitters can reflect both the dysfunctional state of monoaminergic systems and their involvement in adaptation processes. To answer this question, an assessment of the content and activity of enzymes of synthesis and degradation and the expression of receptors is needed [116]. In addition, it is known that the development of mediator systems is associated with the activity of the endocrine system [117] and the immune system [118]. The influence of maternal deprivation on the formation of dysfunction of the hypothalamic–pituitary–adrenal system is well described in the literature and the role of the glucocorticoid system in post-stress disorders is shown. The role of other hormones (i.e., vasopressin, oxytocin) in the formation of a hyperactive endophenotype in response to childhood trauma has been studied to a lesser extent [117,118]. A promising line of research also seems to be the search for biomarkers of neuroinflammation in the brain structures and peripheral tissues of animals exposed to early life stress.

## Figures and Tables

**Table 1:** The weights of male and female rats reared by their mother (Control) or subjected to maternal separation and sibling isolation on postnatal days 2–18 (MSI) at the ages of 1 and 2 months.

			Control, m	MSI, m	Control, f	MSI, f
**Series 1**						
P28		n	10	11	9	10
		weight	33.0 ± 0.7	30.7 ± 2.4	32.3 ± 1.1	31.3 ± 1.8
ANOVA			Sex main effect: F (1, 36) = 0.001, *p* = 0.975; Rearing main effect: F (1, 36) = 0.869, *p* = 0.357			
P56		n	12	16	13	12
		weight	160.6 ± 6.4	149.4 ± 6.2	146.9 ± 7.1	149.0 ± 5.2
ANOVA			Sex main effect: F (1, 36) = 1.636, *p* = 0.207; Rearing main effect: F (1, 36) = 0.289, *p* = 0.594			
**Series 2**						
P58	n		26	24	24	22
	weigh		172.7 ± 6.6	162.5 ± 5.9	159.6 ± 5.8	150.1 ± 5.0
ANOVA			Sex main effect: F (1, 92) = 4.60, *p* = 0.035; Rearing main effect: F (1, 92) = 2.781, *p* = 0.099			
P75	weigh		310.7 ± 7.3	304.9 ± 7.1	**252.9 ± 5.6** #	**232.1 ± 5.0** * #
ANOVA			Sex main effect: F (1, 92) = 9.798, *p* = 0.003; Rearing main effect: F (1, 92) = 4.250, *p* = 0.042			

**Note:** The results are shown for groups in which the levels of monoamines were analyzed. m—male, f—female; P—postnatal day, n—number of animals; *p* < 0.05 in comparison with * the Control; #—corresponding group of males (Newman–Keuls test).

**Table 2:** Norepinephrine levels (nmol/g tissue) in the brain structures in rats subjected to maternal separation on postnatal days 2–18 (MSI) compared with normally reared rats (Control).

1 Month					
		Control, m	MSI, m	Control, f	MSI, f
Frontal cortex		1.79 ± 0.06	1.94 ± 0.08	1.88 ± 0.14	1.89 ± 0.09
Hippocampus		**1.11 ± 0.06**	**1.38 ± 0.05** *	1.19 ± 0.09	1.27 ± 0.07
		Rearing main effect: F (1, 36) = 6.199, *p* = 0.017			
Striatum		2.44 ± 0.24	2.68 ± 0.22	2.49 ± 0.18	1.99 ± 0.25
Hypothalamus		**6.89 ± 0.15**	7.86 ± 0.24 * #	6.91 ± 0.24	6.46 ± 0.20
		Sex main effect: F (1, 36) = 9.798, *p* = 0.003; Sex × Rearing interaction: F (1, 36) = 12.312, *p* = 0.001			
**2 months**					
		Control, m	MSI, m	Control, f	MSI, f
Frontal cortex		2.61 ± 0.12	2.62 ± 0.06	2.77 ± 0.10	2.50 ± 0.12
Hippocampus		**2.22 ± 0.09**	1.94 ± 0.08 *	1.96 ± 0.08 #	1.81 ± 0.08
		Sex main effect: F (1,49) = 4.757.3, *p* = 0.034; Rearing main effect: F (1, 49)= 6.078, *p* = 0.017 * *p* = 0.063			
Striatum		3.40 ± 0.53	4.11 ± 0.32	2.98 ± 0.34	3.00 ± 0.51
Hypothalamus		10.51 ± 0.37	10.61 ± 0.30	**11.47 ± 0.59**	9.61 ± 0.17 *
		Sex × Rearing interaction: F (1, 48) = 4.871, *p* = 0.032			
**3 months (after double hit)**					
		Control, m	MSI, m	Control, f	MSI, f
Frontal cortex	FS	1.82 ± 0.07	2.01 ± 0.07	2.41 ± 0.07 #	2.28 ± 0.08 #
	EmS	1.99 ± 0.07	2.03 ± 0.09	**2.60 ± 0.08 #**	2.38 ± 0.07 * #
	Sex main effect: F (1, 88) = 72.170, *p* < 0.001; Stress main effect: F (1, 88) = 5.150, *p* = 0.010; Sex × Rearing interaction: F (1, 88) = 6.889, *p* = 0.018				
Hippocampus	FS	2.33 ± 0.05	2.22 ± 0.09	2.30 ± 0.09	2.14 ± 0.10
	EmS	**2.66 ± 0.17**	2.26 ± 0.07 *	2.44 ± 0.11	2.34 ± 0.13
	Rearing main effect: F (1, 88)= 6.699, *p* = 0.011; Stress main effect: F (1, 88) = 5.65, *p* = 0.020				
Striatum	FS	4.18 ± 0.47	2.79 ± 0.26	2.33 ± 0.33 #	2.92 ± 0.21
	EmS	3.36 ± 0.20 s	2.59 ± 0.31	2.54 ± 0.28	3.17 ± 0.35
	Sex main effect: F (1, 88) = 4.775, *p* = 0.031; Rearing main effect: F (1, 88) = 14.496, *p* < 0.001				
Hypothalamus	FS	11.01 ± 0.58	10.85 ± 0.46	10.43 ± 0.46	10.45 ± 0.39
	EmS	12.61 ± 0.97	11.19 ± 0.51	9.94 ± 0.55	9.99 ± 0.31 #
	Rearing main effect: F (1, 88) = 8.702, *p* = 0.004; Sex × stress interaction: F (1, 88) = 3.080, *p* = 0.083				

**Note:** m—males, f—females, FS—foot shock, EmS—emotional stress; *p* < 0.05 in comparison with the * Control, # males of the corresponding subgroup; s—in comparison with the effect of FS (Newman–Keuls test).

**Table 3:** The levels of dopamine and its metabolites (nmol/g tissue) in the brain structures of rats subjected to maternal separation on postnatal days 2–18 (MSI) and normally reared rats (Control).

1 Month					
Frontal Cortex					
		Control, m	MSI, m	Control, f	MSI, f
Dopamine		0.71 ± 0.33	0.49 ± 0.12.	0.54 ± 0.11	0.44 ± 0.02
DOPAC		0.22 ± 0.09	0.17 ± 0.02	0.18 ± 0.02	0.15 ± 0.01
HVA		0.43 ± 0.10	0.31 ± 0.04	**0.47 ± 0.07**	0.27 ± 0.02 *
		Rearing main effect: F (1, 36) = 5.923, *p* = 0.020			
DOPAC/DA		0.36 ± 0.03	0.38 ± 0.03	0.35 ± 0.03	0.33 ± 0.02
HVA/DA		0.84 ± 0.11	0.72 ± 0.09	**0.95 ± 0.11**	0.62 ± 0.05 *
		Rearing main effect: F (1, 36) = 5.303, *p* = 0.027			
**Hippocampus**					
		Control, m	MSI, m	Control, f	MSI, f
Dopamine		0.10 ± 0.02	0.12 ± 0.04.	0.15 ± 0.03	0.09 ± 0.02
DOPAC		0.05 ± 0.00	0.05 ± 0.02	0.06 ± 0.03	0.04 ± 0.00
HVA		nd	nd	nd	nd
DOPAC/DA		0.57 ± 0.06	0.48 ± 0.05	0.49 ± 0.05	0.56 ± 0.08
**Striatum**					
		Control, m	MSI, m	Control, f	MSI, f
Dopamine		26.85 ± 2.89	21.02 ± 2.05	23.41 ± 3.41	27.15 ± 2.54
DOPAC		**8.97 ± 0.89**	6.17 ± 0.91 *	4.06 ± 0.53 #	5.43 ± 0.41
		Sex main effect: F (1, 36) = 14.393, *p* < 0.001; Sex × Rearing interaction: F (1, 36) = 7.842, *p* = 0.008			
HVA		**3.88 ± 0.49**	2.63 ± 0.29 *	2.57 ± 0.24 #	2.83 ± 0.18
		Sex × Rearing interaction: F (1, 36) = 5.040, *p* = 0.031			
DOPAC/DA		0.34 ± 0.02	0.30 ± 0.03	0.18 ± 0.01 #	0.20 ± 0.01 #
		Sex main effect: F (1, 36) = 40.406, *p* < 0.001			
HVA/DA		0.15 ± 0.01	0.13 ± 0.01	0.12 ± 0.01	0.11 ± 0.01
		Sex main effect: F (1, 36) = 6.705, *p* = 0.0138; Rearing main effect: F (1, 36) = 3.908, *p* = 0.055			
**Hypothalamus**					
		Control, m	MSI, m	Control, f	MSI, f
Dopamine		**2.35 ± 0.18**	1.71 ± 0.14 *	1.80 ± 0.19	1.87 ± 0.15
		Sex × Rearing interaction: F (1, 36) = 4.673, *p* = 0.037			
DOPAC		0.53 ± 0.05	0.38 ± 0.03	0.43 ± 0.04	0.52 ± 0.05
		Sex × Rearing interaction: F (1, 36) = 8.010, *p* = 0.008			
HVA		0.21 ± 0.04	0.16 ± 0.01	0.13 ± 0.01	0.19 ± 0.01
		Sex × Rearing interaction: F (1, 36) = 6.198, *p* = 0.018			
DOPAC/DA		0.22 ± 0.01	0.23 ± 0.01	**0.24 ± 0.01**	0.28 ± 0.01 *
		Sex main effect: F (1, 36) = 14.823, *p* < 0.001; Rearing main effect: F (1, 36) = 4.976, *p* = 0.032; Sex × Rearing interaction: F (1, 36) = 3.555, *p* = 0.067			
HVA/DA		0.09 ± 0.01	0.10 ± 0.01	0.08 ± 0.01	0.11 ± 0.01
		Rearing main effect: F (1, 36) = 3.384, *p* = 0.074			
**2 months**					
**Frontal cortex**					
		Control, m	MSI, m	Control, f	MSI, f
Dopamine		0.89 ± 0.22	0.84 ± 0.09	0.66 ± 0.04	0.55 ± 0.03
DOPAC		0.19 ± 0.03	0.20 ± 0.02	0.13 ± 0.01	0.12 ± 0.01
HVA		0.29 ± 0.03	0.30 ± 0.03	0.21 ± 0.03	0.22 ± 0.02
		Sex main effect: F (1, 49) = 8.508, *p* = 0.005			
DOPAC/DA		0.23 ± 0.03	0.24 ± 0.03	0.20 ± 0.03	0.22 ± 0.02
		Sex main effect: F (1, 49) = 4.800, *p* = 0.033			
HVA/DA		0.84 ± 0.11	0.72 ± 0.09	0.95 ± 0.11	0.62 ± 0.05
**Hippocampus**					
		Control, m	MSI, m	Control, f	MSI, f
Dopamine		0.16 ± 0.02	0.17 ± 0.03	0.63 ± 0.49	0.14 ± 0.02
DOPAC		0.04 ± 0.01	0.05 ± 0.01	0.14 ± 0.07	0.07 ± 0.01
HVA		nd	nd	nd	nd
DOPAC/DA		0.26 ± 0.02	0.30 ± 0.03	0.48 ± 0.05 #	0.56 ± 0.06
		Sex main effect: F (1, 49) = 41.116, *p* < 0.001			
**Striatum**					
		Control, m	MSI, m	Control, f	MSI, f
Dopamine		37.37 ± 4.19	30.01 ± 3.17	**47.30 ± 3.85**	35.07 ± 3.26 *
		Sex main effect: F (1, 49) = 4.525, *p* = 0.038; Rearing main effect: F (1, 49) = 7.958, *p* = 0.007			
DOPAC		7.97 ± 1.00	6.46 ± 0.64	8.15 ± 0.57	9.12 ± 0.73
		Sex × Rearing interaction: F (1, 49) = 3.626, *p* = 0.063			
HVA		**3.25 ± 0.45**	2.08 ± 0.22 *	3.43 ± 0.28	3.43 ± 0.39
		Sex main effect: F (1, 49) = 5.026, *p* = 0.029; Rearing main effect: F (1, 49) = 3.142, *p* = 0.082; Sex × Rearing interaction: F (1, 49) = 3.900, *p* = 0.054			
DOPAC/DA		0.21 ± 0.01	0.22 ± 0.01	**0.18 ± 0.01 #**	0.27 ± 0.01 * #
		Rearing main effect: F (1, 49) = 19.378, *p* < 0.001; Sex × Rearing interaction: F (1, 49) = 18.895, *p* < 0.001			
HVA/DA		**0.09 ± 0.01**	0.07 ± 0.01 *	0.07 ± 0.01	0.10 ± 0.01
		Sex main effect: F (1, 49) = 8.221, *p* = 0.006; Rearing main effect: F (1, 49) = 4.614, *p* = 0.037; Sex × Rearing interaction: F (1, 49) = 4.568, *p* = 0.037			
**Hypothalamus**					
		Control, m	MSI, m	Control, f	MSI, f
Dopamine		2.40 ± 0.13	2.20 ± 0.09	2.25 ± 0.19	2.15 ± 0.10
DOPAC		0.49 ± 0.03	0.46 ± 0.03	0.56 ± 0.05	0.44 ± 0.02
		Rearing main effect: F (1, 49) = 4.853. *p* = 0.032			
HVA		**0.25 ± 0.01**	0.15 ± 0.01 *	**0.34 ± 0.03 #**	0.22 ± 0.01 * #
		Sex main effect: F (1, 49) = 23.242, *p* < 0.001; Rearing main effect: F (1, 49) = 44.449, *p* < 0.001			
DOPAC/DA		0.21 ± 0.01	0.21 ± 0.01	**0.25 ± 0.01 #**	0.21 ± 0.01 *
		Sex main effect: F (1, 49)= 4.165, *p* = 0.036; Rearing main effect: F (1, 49) = 4.443, *p* = 0.040; Sex × Rearing interaction: F (1, 49) = 6.979, *p* = 0.011			
HVA/DA		**0.11 ± 0.01**	0.07 ± 0.0.01 *	**0.15 ± 0.01 #**	0.11 ± 0.01* #
		Sex main effect: F (1, 49)= 33.821, *p* < 0.001; Rearing main effect: F (1, 49) = 36.799, *p* < 0.001			
**3 months (after double hit)**					
**Frontal Cortex**					
		Control, m	MSI, m	Control, f	MSI, f
Dopamine	FS	1.83 ± 0.52	0.98 ± 0.28	1.45 ± 0.36	1.82 ± 0.47
	EmS	1.43 ±0.43	1.72 ± 0.38	1.18 ± 0.19	1.20 ± 0.25
DOPAC	FS	0.22 ± 0.05	0.16 ± 0.04	0.21 ± 0.04	0.26 ± 0.05
	EmS	0.20 ± 0.07	0.23 ± 0.05	0.16 ± 0.03	0.14 ± 0.02
HVA	FS	0.21 ± 0.03	0.21 ± 0.03	0.22 ±0.04	0.28 ± 0.04
	EmS	0.24 ± 0.06	0.23 ± 0.04	0.19 ± 0.03	0.23 ± 0.02
DOPAC/DA	FS	0.14 ± 0.01	0.17 ± 0.01	0.16 ± 0.01	0.17 ± 0.02
	EmS	0.14 ± 0.01	0.13 ± 0.01 (s)	0.13 ± 0.01	0.13 ± 0.01 s
	Stress main effect: F (1, 88) = 14.194, *p* < 0.001; Rearing × stress interaction: F (1, 88) = 3.781, *p* = 0.055				
HVA/DA	FS	0.18 ± 0.03	0.24 ± 0.03	0.21 ± 0.05	0.21 ± 0.03
	EmS	0.20 ± 0.02	0.16 ± 0.02	0.17 ± 0.02	0.23 ± 0.03
	Sex × Rearing × stress interaction: F (1, 88) = 5.189, *p* = 0.025				
**Hippocampus**					
		Control, m	MSI, m	Control, f	MSI, f
Dopamine	FS	0.17 ± 0.02	0.30 ± 0.05	0.19 ± 0.02	0.28 ± 0.05
	EmS	0.26 ± 0.06	0.27 ± 0.05	0.16 ± 0.01	0.25 ± 0.03
DOPAC	FS	0.04 ± 0.00	0.07 ± 0.01	0.06 ± 0.01	0.06 ± 0.01
	EmS	0.07 ± 0.01	0.08 ± 0.01	0.05 ± 0.00	0.06 ± 0.01
	Rearing main effect: F (1, 88) = 5.294, *p* = 0.015				
DOPAC/DA	FS	0.26 ± 0.01	0.30 ± 0.03	0.30 ± 0.01	0.27 ± 0.03
	EmS	0.31 ± 0.02	0.31 ± 0.04	0.33 ± 0.02	0.25 ± 0.02
	Sex × Rearing interaction: F (1, 88) = 4.532, *p* = 0.036				
**Striatum**					
		Control, m	MSI, m	Control, f	MSI, f
Dopamine	FS	33.67 ± 5.70	31.64± 4.25	47.73 ± 5.38	40.09 ± 7.27
	EmS	43.53 ± 3.94	38.87± 5.38	53.99 ± 4.66	46.29 ± 4.92
	Sex main effect: F (1, 88) = 7.440, *p* = 0.008; Stress main effect: F (1, 88) = 3.982, *p* = 0.041				
DOPAC	FS	3.30 ± 0.55	3.38 ± 0.50	4.47 ± 0.47	3.61 ± 0.64
	EmS	4.53 ± 0.47	4.01 ± 0.57	5.40 ± 0.57	4.38 ± 0.51
	Stress main effect: F (1, 88) = 5.472, *p* = 0.021				
HVA	FS	1.94 ± 0/30	1.87 ± 0.27	2.62 ± 0.26	2.43 ± 0.33
	EmS	2.62 ± 0.26	2.28 ± 0.32	2.83 ± 0.20	3.13 ± 0.39
	Sex main effect: F (1, 88) = 7.780, *p* = 0.006; Stress main effect: F (1, 88) = 5.951, *p* = 0.017				
DOPAC/DA	FS	0.10 ± 0.00	0.11 ± 0.00	0.09 ± 0.00	0.09 ± 0.00
	EmS	0.10 ± 0.00	0.10 ± 0.00	0.10 ± 0.01	0.09 ± 0.00
	Sex main effect: F (1, 88) = 9.319, *p* = 0.003				
HVA/DA	FS	0.07 ± 0.02	0.07 ± 0.01	0.05 ± 0.00	0.09 ± 0.02
	EmS	0.06 ± 0.00	0.06 ± 0.00	0.05 ± 0.00	0.07 ± 0.00
	Sex × Rearing interaction: F (1, 88) = 3.692, *p* = 0.058				
**Hypothalamus**					
		Control, m	MSI, m	Control, f	MSI, f
Dopamine	FS	2.27 ± 0.22	1.92 ± 0.13	2.23 ± 0.41	2.11 ± 0.12
	EmS	2.26 ± 0.26	2.27 ± 0.25	2.48 ± 0.80	1.93 ± 0.12
DOPAC	FS	0.41 ± 0.06	0.39 ± 0.02	0.53 ± 0.09	0.41 ± 0.02
	EmS	0.41 ± 0.05	0.40 ± 0.05	0.49 ± 0.11	0.37 ± 0.02
HVA	FS	0.09 ± 0.01	0.10 ± 0.01	0.25 ± 0.02	0.18 ± 0.02 *
	EmS	0.12 ± 0.01	0.10 ± 0.01	0.21 ± 0.02	0.18 ± 0.02
	Sex main effect: F (1, 88) = 85.001, *p* < 0.001; Rearing main effect: F (1, 88) = 7.048, *p* = 0.009; Sex × Rearing interaction: F (1, 88) = 3.048, *p* = 0.084				
DOPAC/DA	FS	0.18 ± 0.01	0.21 ± 0.01	0.24 ± 0.01	0.20 ± 0.01
	EmS	0.18 ± 0.01	0.18 ± 0.01	0.22 ± 0.01	0.20 ± 0.01
	Sex main effect: F (1, 88) = 17.931, *p* < 0.001;Rearing main effect: F (1, 88) = 3.259, *p* = 0.074; Stress main effect: F (1, 88) = 4.076, *p* = 0.046; Sex × Rearing interaction: F (1, 88) = 12.503, *p* < 0.001; Sex × Rearing × stress interaction: F (1, 88) = 2.962, *p* = 0.088				
HVA/DA	FS	0.04 ± 0.00	0.05 ± 0.01	0.13 ± 0.01	0.09 ± 0.01
	EmS	0.06 ± 0.00	0.04 ± 0.00	0.12 ± 0.01	0.10 ± 0.01
	Sex main effect: F (1, 88) = 76.719, *p* < 0.001; Rearing main effect: F (1, 88) = 3.364, *p* = 0.023; Sex × Rearing interaction: F (1, 88) = 12.503, *p* < 0.001				

**Note:** m—males, f—females, FS—foot shock, EmS—emotional stress; *p* < 0.05 in comparison with the * Control, #—males of the corresponding subgroup; s—*p* < 0.05; (s)—*p* < 0.1 in comparison with the effect of FS (Newman–Keuls test).

**Table 4:** The levels of 5-hidpoxytriptamine and its metabolite (nmol/g tissue) in the brain structures of rats subjected to maternal separation on postnatal days 2–18 (MSI) and normally reared rats (Control).

1 Months					
Frontal Cortex					
		**Control, m**	**MSI, m**	**Control, f**	MSI, f
5-HT		2.50 ± 0.01	2.42 ± 0.06	2.62 ± 0.09	2.53 ± 0.04
HIAA		2.00 ± 0.13	1.65 ± 0.18	1.87 ± 0.08	1.79 ± 0.08
HIAA/5-HT		0.8 ± 0.03	0.68 ± 0.07	0.72 ± 0.04	0.71 ± 0.04
**Hippocampus**					
		Control, m	MSI, m	Control, f	MSI, f
5-HT		2.25 ± 0.11	2.94 ± 0.40	2.71 ± 0.14	2.86 ± 0.09
		Rearing main effect: F (1, 36) = 4.175, *p* = 0.034			
HIAA		2.24 ± 0.10	2.56 ± 0.12	2.71 ± 0.14	2.57 ± 0.10
		Sex main effect: F (1, 36) = 4.271, *p* = 0.046; Sex × Rearing interaction: F (1, 36) = 4.010, *p* = 0.053			
HIAA/5-HT		1.00 ± 0.0	0.93 ± 0.06	1.01 ± 0.06	0.90 ± 0.02
**Striatum**					
		Control, m	MSI, m	Control, f	MSI, f
5-HT		2.06 ± 0.07	2.26 ± 0.17	2.79 ± 0.20 #	2.82 ± 0.20 #
		Sex main effect: F (1, 36) = 15.365, *p* < 0.001			
HIAA		4.55 ± 0.19	4.47 ± 0.27	4.80 ± 0.16	4.53 ± 0.31
HIAA/5-HT		2.23 ± 0.11	2.06 ± 0.18	1.79 ± 0.13 #	1.62 ± 0.06
		Sex main effect: F (1, 36) = 12.703, *p* = 0.001			
**Hypothalamus**					
		Control, m	MSI, m	Control, f	MSI, f
5-HT		**6.25 ± 0.13**	5.58 ± 0.22 *	5.46 ± 0.20 #	5.34 ± 0.12
		Sex main effect: F (1, 36) = 9.347, *p* = 0.004; Rearing main effect: F (1, 36) = 5.467, *p* = 0.025			
HIAA		**5.30 ± 0.19**	4.48 ± 0.28 *	4.97 ± 0.11	5.18 ± 0.23
		Sex × Rearing interaction: F (1, 36) = 5.624, *p* = 0.023			
HIAA/5-HT		0.85 ± 0.03	0.79 ± 0.03	0.92 ± 0.03	0.97 ± 0.03
**2 months**					
**Frontal cortex**					
		Control, m	MSI, m	Control, f	MSI, f
5-HT		**2.78 ± 0.08**	3.28 ± 0.09 *	**3.11 ± 0.10 #**	2.41 ± 0.15 * #
		Sex main effect: F (1, 49) = 5.980, *p* = 0.018; Sex × Rearing interaction: F (1, 49) = 32.555, *p* < 0.001			
HIAA		**1.38 ± 0.06**	1.62 ± 0.06 *	**1.44 ± 0.05**	1.05 ± 0.06 *
		Sex main effect: F (1, 49) = 18.435, *p* < 0.001; Sex × Rearing interaction: F (1, 49) = 31.328, *p* < 0.001			
HIAA/5-HT		0.5 ± 0.02	0.49 ± 0.01	0.47 ± 0.02	0.44 ± 0.02
		Sex main effect: F (1, 49) = 6.116, *p* = 0.017			
**Hippocampus**					
		Control, m	MSI, m	Control, f	MSI, f
5-HT		3.37 ± 0.08	3.37 ± 0.27	2.74 ± 0.14	2.59 ± 0.11
		Sex main effect: F (1, 49) = 17.219, *p* < 0.001			
HIAA		1.67 ± 0.05	1.66 ± 0.06	1.77 ± 0.10	1.86 ± 0.06
		Sex main effect: F (1, 49) = 4.856, *p* = 0.033			
HIAA/5-HT		0.50 ± 0.02	0.51 ± 0.03	0.66 ± 0.04	0.72 ± 0.02
		Sex main effect: F (1, 49) = 55.357, *p* < 0.001			
**Striatum**					
		Control, m	MSI, m	Control, f	MSI, f
5-HT		2.70 ± 0.18	2.92 ± 0.14	2.45 ± 0.13 #	2.42 ± 0.13 #
		Sex main effect: F (1, 49) = 5.210, *p* = 0.027			
HIAA		3.55 ± 0.15	3.87 ± 0.19	3.51 ± 0.14	3.85 ± 0.22
		Rearing main effect: F (1, 49) = 3.577, *p* = 0.064			
HIAA/5-HT		1.37 ± 0.11	1.35 ± 0.06	1.49 ± 0.10 #	1.64 ± 0.12 #
		Sex main effect: F (1, 49) = 4.094, *p* = 0.048			
**Hypothalamus**					
		Control, m	MSI, m	Control, f	MSI, f
5-HT		**6.81 ± 0.45**	5.95 ± 0.19 *	6.51 ± 0.45 #	6.46 ± 0.13
		Sex × Rearing interaction: F (1, 49) = 3.289, *p* = 0.075			
HIAA		**5.30 ± 0.19**	4.48 ± 0.28 *	4.97 ± 0.11	5.18 ± 0.23
		Sex main effect: F (1, 49)= 10.471, *p* = 0.002; Rearing main effect: F (1, 49) = 6.302, *p* = 0.015			
HIAA/5-HT		0.58 ± 0.02	0.57 ± 0.02	**0.69 ± 0.02**	0.63 ± 0.02 * #
		Sex main effect: F (1, 49) = 22.276, *p* < 0.001; Rearing main effect: F (1, 49) = 4.053, *p* = 0.050; Sex × Rearing interaction: F (1, 49) = 3.686, *p* = 0.060			
**3 months (after double hit)**					
**Frontal Cortex**					
		Control, m	MSI, m	Control, f	MSI, f
5-HT	FS	3.51 ± 0.14	3.37 ± 0.14	3.70 ± 0.19	3.71 ± 0.13
	EmS	3.21 ± 0.13	3.59 ± 0.13	3.62 ± 0.09	3.86 ± 0.09
	Sex main effect: F (1, 88) = 9.933, *p* = 0.002; Rearing × stress interaction: F (1, 88) = 3.707, *p* = 0.057				
HIAA	FS	1.63 ± 0.09	1.43 ± 0.09	1.66 ± 0.05	1.43 ± 0.09
	EmS	1.64 ± 0.10	1.63 ± 0.07	1.60 ± 0.07	1.44 ± 0.08
	Rearing main effect: F (1, 88) = 6.884, *p* = 0.010				
HIAA/5-HT	FS	0.46 ± 0.01	0.42 ± 0.02	0.46 ± 0.02	0.39 ± 0.03
	EmS	0.51 ± 0.02	0.46 ± 0.01	0.44 ± 0.02	0.38 ± 0.02
	Sex main effect: F (1, 88) = 10.51, *p* = 0.002; Rearing main effect: F (1, 88) = 15.93, *p* < 0.001; Sex ×stress interaction: F (1, 88) = 3.32, *p* = 0.072				
**Hippocampus**					
		Control, m	MSI, m	Control, f	MSI, f
5-HT	FS	2.67 ± 0.26	2.96 ± 0.14	2.80 ± 0.26	3.48 ± 0.40
	EmS	2.82 ± 0.10	3.21 ± 0.13	2.86 ± 0.25	2.95 ± 0.11
	Rearing main effect: F (1, 88) = 5.156, *p* = 0.026				
HIAA	FS	1.42 ± 0.04	1.42 ± 0.06	1.78 ± 0.06	2.01 ± 0.26
	EmS	1.74 ± 0.06 s	1.62 ± 0.06	1.87 ± 0.13	1.78 ± 0.05
	Sex main effect: F (1, 88) = 16.510, *p* < 0.001 Sex × stress interaction: F (1, 88) = 4.63, *p* = 0.034				
HIAA/5-HT	FS	0.57 ± 0.04	0.49 ± 0.02	0.69 ± 0.05	0.60 ± 0.05
	EmS	0.62 ± 0.02	0.51 ± 0.02	0.67 ± 0.03	0.61 ± 0.01
	Sex main effect: F (1, 88) = 16.759, *p* < 0.001; Rearing main effect: F (1, 88) = 14.452, *p* < 0.001				
**Striatum**					
		Control, m	MSI, m	Control, f	MSI, f
5-HT	FS	4.07 ± 0.20	3.53 ± 0.18	3.66 ± 0.17	4.15 ± 0.25
	EmS	3.82 ± 0.18	3.61 ± 0.20	3.54 ± 0.14	4.44 ± 0.29
	Sex × Rearing interaction: F (1, 88) = 13.859, *p* < 0.001				
HIAA	FS	2.99 ± 0.16	2.46 ± 0.14	3.08 ± 0.10	2.82 ± 0.15
	EmS	3.07 ± 0.17	2.59 ± 0.16	2.86 ± 0.15	3.02 ± 0.19
	Rearing main effect: F (1, 88) = 6.273, *p* = 0.014; Sex × Rearing interaction: F (1, 88) = 4.259, *p* = 0.042				
HIAA/5-HT	FS	0.74 ± 0.03	0.70 ± 0.03	0.86 ± 0.04	0.69 ± 0.02
	EmS	0.80 ± 0.03	0.72 ± 0.02	0.82 ± 0.04	0.69 ± 0.02
	Rearing main effect: F (1, 88) = 24.97, *p* < 0.001; Sex × Rearing interaction: F (1, 88) = 3.967, *p* = 0.049				
**Hypothalamus**					
		Control, m	MSI, m	Control, f	MSI, f
5-HT	FS	6.13 ± 0.30	5.59 ± 0.20	5.62 ± 0.23	6.19 ± 0.18
	EmS	6.81 ± 0.61	5.88 ± 0.24	5.16 ± 0.28	6.02 ± 0.16
	Sex × Rearing interaction: F (1, 88) = 10.115, *p* = 0.002; Sex × stress interaction: F (1, 88) = 3.110, *p* = 0.081				
HIAA	FS	2.83 ± 0.13	2.50 ± 0.11	3.14 ± 0.10	3.05 ± 0.08
	EmS	3.35 ± 0.25	2.75 ± 0.13 s	2.92 ± 0.15	3.11 ± 0.09
	Rearing main effect: F (1, 88) = 3.889, *p* = 0.052; Sex × Rearing interaction: F (1, 88) = 6.165, *p* = 0.015; Sex × stress interaction: F (1, 88) = 5.110, *p* = 0.026				
HIAA/5-HT	FS	0.47 ± 0,02	0.45 ± 0.01	0.57 ± 0.02	0.50 ± 0.02
	EmS	0.50 ± 0.01	0.47 ± 0.01	0.57 ± 0.01	0.52 ± 0.02
	Sex main effect: F (1, 88) = 35.432, *p* < 0.001; Rearing main effect: F (1, 88) = 13.028, *p* < 0.001; Stress main effect: F (1, 88) = 2.978, *p* = 0.088				

**Note:** m—males, f—females, FS—foot shock, EmS—emotional stress; *p* < 0.05 in comparison with the *—control, #—males of the corresponding subgroup; ^s^*—p* < 0.05.

**Table 5:** The levels of monoamines in the brain structures of rats exposed to MSI on P2–P18 and control animals.

ma	NE		DA		DOPAC		HVA		DOPAC/DA		HVA/ DA		5-HT		HIAA		HIAA/ 5-HT	
Sex	m	f	m	f	m	f	m	f	m	f	m	f	m	f	m	f	m	f
	**1 month**																	
FC	=	=	=	=	=	=	**↓**	**↓ ** *	=	=	**↓**	**↓ ** *	=	=	=	=	=	=
Hip	**↑ ** *	**↑**	=	=	=	=	nd	nd	=	=	=	=	↑	**↑**	=	=	=	=
Str	=	=	=	=	**↓ ** *	=	**↓ ** *	**=**	=	=	↓	↓	=	=	=	=	=	=
Hyp	↑ *	=	↓ *	=	=	**=**	=	**=**	**↑**	**↑ ** *	**↑**	**↑**	**↓ ** *	**↓**	**↓ ** *	=	=	=
	**2 months**																	
FC	=	=	=	=	=	=	=	=	=	=	=	=	**↑ ** *	**↓ ** *	**↑ ** *	**↓ ** *	=	=
Hip	**↓ ** *	**↓**	=	=	=	=	=	=	=	=	=	=	=	=	=	=	=	=
Str	=	=	↓	**↓ ** *			**↓ ** *	=	↑	↑ *	**↓ ** *	=	=	=	↑	↑ *	=	=
Hyp	=	**↓ ** *	=	=	↓	↓	↓ *	↓ *	=	**↓ ** *	**↓ ** *	**↓ ** *	**↓ ** *	**=**	**↓ ** *	=	**=**	↓ *

**Note:** ma—monoamine; m—male, f—female; FC—frontal Cortex, Hip—hippocampus, Str—striatum, Hyp—hypothalamus; ↑ and ↓ show increase and decrease in monoamine level in comparison with the corresponding control; ANOVA—yellow indicates Rearing main effect, blue and rose—Sex × Rearing interaction, *—*p* < 0.05, post hoc Newman–Keuls test.

## Data Availability

Data are contained within the article.

## Abbreviations

P: post-natal day; MSI: maternal separation and sibling isolation; FS: foot shock; EmS: emotional shock; aOF: automatic open field; cOF: classic open field with visual control; EPM: elevated plus maze; SIT: social interaction test; TCT: three-chamber social test; FC: frontal cortex: Hip: hippocampus; Str: striatum; Hyp: hypothalamus; NE: norepinephrine; DA: dopamine; DOPAC: 3:4-dihydroxyphenylacetic acid; HVA: homovanillic acid; 5-HT: 5-hydroxytryptamine; 5-HIAA: 5-hydroxyindole acetic acid.
